# The mis-measurement of extreme global poverty: A case study in the Pacific Islands

**DOI:** 10.1177/1440783314523867

**Published:** 2015-09

**Authors:** Christopher Deeming, Bina Gubhaju

**Affiliations:** University of Bristol, UK; Australian National University, Australia

**Keywords:** child welfare, development, household consumption, quantitative analysis, research methods, social problems, social welfare, standard of living

## Abstract

Debate over the measurement of global poverty in low- and middle-income countries continues unabated. There is considerable controversy surrounding the ‘dollar a day’ measure used to monitor progress against the Millennium Development Goals. This article shines fresh light on the debate with new empirical analyses of poverty (including child poverty), inequality and deprivation levels in the Pacific island state of Vanuatu. The study focuses not only on economic and monetary metrics and measures, but also the measures of deprivation derived from sociology in relation to shelter, sanitation, water, information, nutrition, health and education. Until recently, there had been few, if any, attempts to study poverty and deprivation disparities among children in this part of the world. Different measures yield strikingly different estimates of poverty. The article, therefore, attempts to situate the study findings in the broader international context of poverty measurement and discusses their implications for future research and the post-2015 development agenda.

This article sheds fresh light on the measurement of global poverty debate with new empirical analyses of poverty, particularly childhood poverty, inequality and deprivation levels in the Pacific island state of Vanuatu. The study focuses not only on economic and monetary measures of poverty drawing on family budget survey data, but also the sociological measures of deprivation and child poverty developed by Peter Townsend and colleagues in relation to shelter, sanitation, water, information, nutrition, health and education (Gordon et al., 2003). Until recently, there had been few, if any, attempts to study poverty and deprivation disparities among children in this part of the world. Here we draw on some of our recent work in this region, commissioned by UNICEF (2011), and some of the data collected within the Global Study on Child Poverty and Disparities framework (UNICEF, 2005, 2007).^1^ The Global Study argues that children experience poverty more acutely than adults because of their vulnerability because of age and dependency and because lost opportunities in childhood often cannot be recaptured in later life.

The article is structured as follows: the next section looks at the measurement of global poverty debate and the successes and failures of the original Millennium Development Goals (MDGs), with the goal of eradicating ‘extreme poverty’, and some of the controversy that now surrounds their replacement post-2015. Next we provide a brief overview of the Vanuatu context, before moving on to discuss the methodologies used to estimate poverty in this inquiry. The study findings are discussed in the fourth section, before the article concludes by discussing the implications of these findings for the global measurement of poverty and policy for the eradication of extreme poverty.

## Debates on the measurement of global poverty

How many people are poor in the world today? This simple question, perhaps unsurprisingly, is extremely difficult to answer, as Carr-Hill (2010) argues. The World Bank has attempted to provide an answer. Since 1990, it has published comprehensive estimates of ‘extreme poverty’, which are now widely accepted by the international community. Subsequently, the Bank’s figures are employed to monitor and appraise global policy and poverty reduction strategies (World Bank, 2001). This is, of course, the well-known international poverty line of US $1 a day, used to monitor progress against the MDG goal of reducing extreme poverty in low- and middle-income countries (LMIC) (Chen and Ravallion, 2012). The number of people in the world estimated to be living on less than a dollar a day – recently adjusted to be $1.25 per day in light of updated global price data – is shown in Table 1. The table also includes estimates for the Pacific Region.

**Table 1.** Global poverty estimates, the number of people (in millions) living below $1.25 a day (2005 purchasing power parity or PPP).

Region	1981	1984	1987	1990	1993	1996	1999	2002	2005	2008
**East Asia and Pacific**	1,096.5	970.0	847.6	926.4	870.8	639.7	655.6	523.1	332.1	284.4
**China**	835.1	719.9	585.7	683.2	632.7	442.8	446.3	363.1	211.9	173.0
**Eastern Europe and Central Asia**	8.2	6.9	6.8	8.9	13.7	18.2	17.8	10.6	6.3	2.2
**Latin America and the Caribbean**	43.3	52.9	49.3	53.4	52.5	53.6	60.1	62.7	47.6	36.8
**Middle East and North Africa**	16.5	15.1	14.6	13.0	11.5	12.3	13.6	12.0	10.5	8.6
**South Asia**	568.4	573.8	593.0	617.3	631.9	630.8	619.5	640.5	598.3	570.9
**Sub-Saharan Africa**	204.9	239.1	256.8	289.7	330.0	349.4	376.8	390.4	394.9	386.0
**Total**	1937.8	1857.7	1768.2	1908.6	1910.3	1704.0	1743.4	1639.3	1389.6	1289.0

*Source*: Chen and Ravallion (2012: 5).

The Bank’s work has been ground-breaking, but critics argue that its estimates are flawed (e.g. Reddy and Pogge, 2010; Townsend, 2002; Wade, 2007). They argue that the Bank’s poverty line appears arbitrary, observing that the calculations underpinning it are not sufficiently anchored to any specification of human requirements. The bias in the Bank’s calculations may be substantial and therefore the extent of global poverty may well be understated.

In addition, the poverty literature in the past decade has placed increased emphasis on the multidimensionality of the concept and measurement of poverty. A burgeoning literature has increasingly questioned the utility of one-dimensional measures of monetary poverty and emphasized the need to examine non-monetary forms of disadvantage and deprivation for families, adults and children (Alkire and Foster, 2010; Minujin and Nandy, 2012). This is particularly important when examining poverty in the Pacific − where absolute poverty has been thought to be less of an issue, but where hardship resulting from limited access to transport, safe water supply, adequate sanitation, health care and education is widely perceived to exist (Abbott and Pollard, 2004; Seniloli, 2005). Deprivation poverty measurement, pioneered by British sociologist Peter Townsend (Rosenfeld, 2010), is particularly relevant to the Pacific island context. In Vanuatu, no government social security programmes operate; thus, high levels of subsistence home-production for survival are prevalent, as discussed below. Defining poverty only in terms of levels of cash income would arguably be inappropriate and problematic in this and similar contexts.

Our article is timely. There is a growing debate, involving nation states and international non-governmental organizations (NGOs), about the successes and failures of the original MDGs and the controversy around their replacement. Despite reductions in ‘extreme poverty’ rates, over 1 billion people in the world live on less than $1.25 per day, as we saw in Table 1. A new international consensus, under the auspices of the United Nations, is emerging to keep up the momentum on the need to end ‘extreme poverty’, forming part of the Post-2015 Development Agenda (UN, 2013).^2^ Recently, the World Bank has (re)set the target for ending extreme poverty in the world within a generation, by 2030. It had set a similar, arguably the same, target back in 1973 when it pledged to eradicate ‘absolute poverty’ in LMICs by the end of the century (i.e. by the year 2000).^3^ Increasingly, policymakers and NGOs are also looking to address the growing problem of inequality in LMICs as part of the post-2015 agenda (Save the Children, 2012; UN, 2013). In this article, we therefore pursue a range of different measures and indicators of poverty and deprivation to develop a more robust analysis of the social and economic challenges facing developing countries and Small Island Developing States (SIDS) like Vanuatu. The use of alternative measures is a way of ‘triangulating’ poverty findings (Rio Group, 2006). By using multiple methodologies, the similarities and differences in results can be seen as validating common findings, but also indicating that some conclusions are dependent on the specific method used. In addition to money metric poverty lines and measures for ‘basic needs’ and ‘extreme poverty’, we consider non-monetary measures of deprivation poverty (UN, 2005). Further, with the inclusion and growing importance of inequality in social policy for development, we adopt some of the inequality poverty measures typically used in high-income countries, such as the median income measures (Atkinson and Marlier, 2010; OECD, 2008, 2011).

## The Pacific Island state of Vanuatu

Vanuatu, which gained its independence in 1980 after 74 years of joint rule by Britain and France, is a Pacific Island Country (PIC) in the Melanesian group. It comprises approximately 80 islands that stretch longitudinally over 650 km of ocean. In all, 65 of the islands are inhabited. The country has six provinces: Tafea, Shefa, Malampa, Penama, Sanma and Torba, and two urban centres. The capital, Port Vila, is located in Shefa province and the other urban centre, Luganville, is located on Espiritu Santo in Sanma province (ADB, 2009). Torba (the northernmost province) and Tafea (the southernmost province) contain some of the most remote islands and atolls.

The population of almost 250,000 people is largely rural (80%); most households rely on agriculture and fishing for their livelihoods (VNSO, 2009b, 2009a). In 2006, less than 20% of the population aged 15 years and over were working for wages and salaries. Unemployment stood at 10% of the adult population, but a significant proportion of the population (40%) grew and produced their own subsistence agriculture products to support themselves and their own families. Around 61% of wage and salary earners lived in urban areas. Vanuatu has no government social security programmes, apart from a provident scheme for public servants and those in the formal labour market. As a result, family and community ties have traditionally provided social safety nets for the most disadvantaged and vulnerable (ADB, 2002).

Vanuatu’s GDP (gross domestic product) per capita in purchasing power terms in 2008 was US $4084 − a little lower than Indonesia – and around 115th in the world (UNDP, 2010). From 2003, Vanuatu has been one of the fastest growing Pacific economies, driven primarily by tourism, construction and aid inflows. Although agriculture accounts for 14.4% of GDP, it is the backbone of household subsistence, with about 80% of the population living in agricultural households (VNSO, 2006). The economy is small and open, with few trade restrictions. The bulk of export earnings come from tourism and the fishing industry. Official Development Assistance is significant, and accounts for more than 5% of GDP. The island is extremely vulnerable to natural disasters, including earthquakes, cyclones, tsunamis and volcanic eruptions. In 2012 Vanuatu ranked 124th (out of 187 countries) on the United Nations Human Development Index (HDI) according to three basic aspects of human development relating to life expectancy, education and income indices. Australia, on the other hand, is in second place behind Norway (UNDP, 2013).^4^

## Methods, empirical strategy and data

In this study child poverty estimates, and disparities, are considered using four different measures of poverty:

- the international ‘dollar a day’ poverty line (i.e. the value used in MDG Goal 1);
- country-level national food and basic needs poverty lines (BNPLs), calculated at the national average level for Vanuatu, but also BNPLs specific to the different regions and localities (these poverty lines are expressed in the national currency, the Vanuatu Vutu [VUV]);
- deprivation poverty, in relation to shelter, sanitation, water, information, nutrition, health and education; and
- relative poverty lines using various percentiles of median equivalized household consumption expenditure.

We derive national and international monetary poverty lines using data from the latest Household Income and Expenditure Survey (HIES) for Vanuatu conducted in 2006 (VNSO, 2006). The HIES sampled 10% of all households, containing close to 20,000 people, with an overall response rate of 84.6%. A two-stage sampling method was used. The first stage involved the selection of Enumeration Areas (EA) using probability proportional to size (PPS) sampling. The size measure was the estimated number of households in the EA, based on 2006 population estimates, noting that some EAs were excluded from the sample frame. The second stage adopted systematic sampling from a list of all households contained in the EA, with lists prepared by interviewers during their visits to the EA. A sample size of 4590 households was adopted for the survey, representing around 10% of the total households in Vanuatu.

A total of 3885 out of the 4590 selected households fully responded to the survey, giving an overall response rate of 84.6%. Only 4% did not fully respond or provided inadequate information to be included in the survey. However 11.4% of households were reported as vacant dwellings which, most probably, include some households that could not be contacted during the survey period. The survey coverage included only persons living in private households during the survey period, September to November 2006. Those living in institutions, such as school dormitories, hospital wards, hostels, and prisons (as well as those households which had temporarily vacated their dwellings), are not captured by the survey. Importantly, the survey estimated the cash value of ‘home-grown’ production at the household level which is included in all of the study expenditure poverty line calculations (VNSO, 2006).

In this study, the calculations of deprivation poverty are based upon data from the Vanuatu Multiple Indicator Cluster Survey (MICS) conducted in 2007, as part of the third round of MICS surveys (MICS3) (GoV/Ministry of Health/UNICEF, 2009). The Global Study on Child Poverty and Disparities, carried out in 53 countries and seven regions since 2007, uses MICS, Demographic and Health surveys (DHS) and other data, to analyse poverty and disadvantages experienced by families with children. Until our recent report for UNICEF, no Pacific island countries had been included in the Global Study (UNICEF, 2011). The MICS surveys provide up-to-date information for assessing the situation of children and women, and much of the data needed for monitoring progress towards goals established by the MDGs and the goals of A World Fit for Children (WFFC) (UNDP, 2009). The Vanuatu MICS survey was conducted as part of the third round of MICS surveys (MICS3), carried out around the world in more than 50 countries, mostly in 2005–6, following the first two rounds of MICS surveys conducted in 1995 and 2000.

The 2007 Vanuatu MICS3 survey covered a nationally representative sample of 2632 households, containing 13,500 people, including 2692 female respondents aged 15–49 years, 6134 children under 18 (including 1634 children under 5). Data were obtained using three questionnaires: the Household Questionnaire, the Individual Questionnaire for Women aged 15–49 and the Questionnaire for Children under 5 years of age. National, and urban and rural estimates were obtained by combining these provincial data. The fieldwork began in November 2007 and concluded in December 2007 (GoV/Ministry of Health/UNICEF, 2009).

Deprivation of basic human need in this study has been measured along seven dimensions, as shown in Table 2. Deprivation is classified as ‘severe’ or ‘less severe’. For example, the severe level of education deprivation is that a child has never attended school, while the less severe measure is that a child is not currently attending school (see Table 2 for definitions).

**Table 2.** Dimensions and definitions of deprivation.

**1. Shelter**
• *Severe* Children aged 0–17 living in a dwelling with no floor material (i.e. earth/sand floor).
• *Less severe* Children aged 0–17 living in a house with inadequate roofing (i.e. thatch/palm leaf).
**2. Sanitation facilities**
• *Severe* Children aged 0–17 with no access to sanitation facilities including a toilet facility of any kind.
• *Less severe* Children using poor sanitation facilities (such as pour flush latrines, covered pit latrines, open pit latrines and buckets).
**3. Safe drinking water**
• *Severe* Children aged 0–17 using unsafe drinking water (open water or surface water).
• *Less severe* Children aged 0–17 using water from an improved source such as communal water (open wells or shared piped water).
**4. Information**
• *Severe* Children aged 3–17 with no access to a radio, television, landline telephone or mobile (i.e. forms of media, information on access to newspapers or computer is not available in the Vanuatu data).
• *Less severe* Children aged 3–17 with no access to a radio or television (i.e. broadcast media).
**5. Food**
• *Severe* Malnutrition of children aged 5 who are more than 3 standard deviations below the international reference populations for stunting or wasting or underweight.
• *Less severe* Diet of poor nutritional value for children aged 0–5 who are more than 2 standard deviations below the international reference for stunting or wasting or underweight.
**6. Education**
• *Severe* Children aged 7–17 who have received no primary or secondary education.
• *Less severe* Children aged 7–17 currently not attending school but who have attended primary education.
**7. Health**
• *Severe* Children aged 1–2 with no immunization against any diseases.
• *Less severe* Children aged 1–2 with some but not all of the recommended vaccinations (bcg, dpt1, dpt2, dpt3, polio0, polio1, polio2, polio3, and measles).

*Source*: Adapted from Gordon et al. (2003: 8).

## Findings: poverty estimates and disparities

### The international dollar a day poverty line

As discussed at the beginning of the article, the global assessment of ‘absolute’ or ‘extreme’ poverty is usually made through the estimation of the number of people living on less than a dollar a day, the very basis of Goal 1 of the MDGs (UNDP, 2009). Table 3 shows the proportion of children (under 17 years of age) in Vanuatu living below the $1.25 a day poverty line. For Vanuatu as a whole, 5.4% of children were below the global poverty line. However, ‘extreme’ poverty is not evenly distributed by geographical region. On the basis of the $1.25 a day line, poverty rates for children range from around 1% in Luganville and Sanma to 12% in Tafea and just over 14% in Torba.

**Table 3.** Children living on less than $1.25 per day.

Region	Total no. children	No. below poverty line	% below poverty line
**Torba**	4,420	628	14.2
**Sanma (rural)**	11,673	123	1.1
**Penama**	15,059	244	1.6
**Malampa**	13,081	307	2.3
**Shefa (rural)**	12,067	1,294	10.7
**Tafea**	15,786	1,867	11.8
**Luganville**	4,408	46	1.0
**Port Vila**	11,492	232	2.0
**Vanuatu**	**87,986**	**4,740**	**5.4**

### Food and basic needs poverty lines

We estimate the incidence of poverty using the BNPL, a money metric poverty line determined from the minimum costs of providing food, clothes and shelter. The calculations were made in the national currency, the VUV, with 100 Vatu being equal to approximately US $1. First, a food poverty line (FPL) is determined from the cost of a minimally nutritious, low-cost diet delivering 2100 kilo calories per person per day (VNSO, 2008).^5^ A budget for non-food items and expenditure is added, for housing, transport, education, clothing, utilities. Thus, an overall basic needs living standard is determined. The poverty line is adjusted using the UNDP (UN Development Programme) equivalence scale to reflect the different needs of households with different numbers of adults and children (Table 4).^6^

**Table 4.** Monthly adult equivalent per capita poverty lines in Vanuatu VUV with children aged 17 or under counted as half an adult.^α^

	FPL A	Non-food basic needs factor B	Estimated non-food expenditure C = A*B	BNPL D = A + C	Monthly cost per household lowest three deciles
**Vanuatu average**	3,064	0.5	1,651	4,716	21,692
**Rural**	2,589	0.3	777	3,366	14,809
**Luganville**	3,594	0.7	2,516	6,110	26,883
**Port Vila**	5,034	1.2	6,041	11,075	53,159

*Source*: VNSO (2008).

α Per Adult Equivalent (PAE) calculations are made using the standard UNDP equivalent scale. Here adult equivalents are derived from equivalence factors thus a household with two adults and two children would be equivalent to three adult equivalents. Vanuatu Vatu (100 Vatu is approximately US $1).

Separate FPLs were calculated for three areas (rural, Port Vila, Luganville) and estimated from actual food expenditure patterns recorded in survey diaries for households in the lowest three deciles of expenditure. Non-food budgets varied according to geography; rural areas having smaller differences between food and non-food expenditure than urban areas. The measured proportions of non-food to food expenditure were taken as the basis for the BNPL non-food factor; applying these actual expenditure amounts to the FPL provides the non-food basic needs factors as illustrated in Table 4. This table also summarizes the monthly per capita adult equivalent poverty lines expressed in Vatu with children under 17 years counted as half an adult.

Before turning to new estimates of child poverty, it is useful to summarize earlier findings for poverty levels for all households, with and without children. An analysis of the 2006 HIES found that, overall, 6% of households (representing 7.4% of the population) did not consume enough to meet basic food needs. Nationally, about 12.9% of households (representing 15.9% of the population) had consumption below the BNPL. In Port Vila 27.2% of households were below the BNPL.

Estimates of child poverty using the Vanuatu-specific BNPL varied according to whether the poverty line is set at the national level, or whether poverty lines were disaggregated by the regional location in which households lived. About 17% of children in Vanuatu were living in households below the BNPL in 2006 (Table 5). About 40% of children in Torba and 25% in Tafea and Shefa lived in poverty. Luganville and Port Vila had the lowest child poverty rates with about 5% of children in these regions in poverty.

**Table 5.** Children under the BNPL.

Region	Total no. children	No. below poverty line	% below poverty line
**Torba**	4,420	1,747	39.5
**Sanma (rural)**	11,673	1,470	12.6
**Penama**	15,059	2,573	17.1
**Malampa**	13,081	1,445	11.0
**Shefa (rural)**	12,067	2,725	22.6
**Tafea**	15,786	4,099	26.0
**Luganville**	4,408	216	4.9
**Port Vila**	11,492	678	5.9
**Vanuatu**	**87,986**	**14,953**	**17.0**

Table 6 shows the proportion of children under the sub-national BNPLs, which include the regional non-food factors described above. In Torba, the figure is now one-quarter of all children living in poverty compared to the 40% under the national poverty line. In Luganville child poverty was twice the level of the national standard (at 11% from 5%) and in Port Vila it is now 33%, up from 6% using the national standard.

**Table 6.** Children under the sub-national BNPL.

Region	Total No. children	No. below poverty line	% below poverty line
**Torba**	4,420	1,068	24.2
**Sanma (rural)**	11,673	544	4.7
**Penama**	15,059	917	6.1
**Malampa**	13,081	465	3.6
**Shefa (rural)**	12,067	1,870	15.5
**Tafea**	15,786	2,784	17.6
**Luganville**	4,408	501	11.4
**Port Vila**	11,492	1,874	16.3
**Vanuatu**	**87,986**	**10,023**	**11.4**

## Deprivation

Although monetary measures of poverty focus on an important dimension of well-being, they only paint a partial picture of the circumstances of disadvantaged families. Income, for example, is a measure of the resources available to households, while expenditures can be thought of as what households achieve with their resources. In addition, it is important to understand the qualitative outcomes households achieve (Ringen, 1988). For example, two households may have similar income levels, but varying living standards because of access to basic services in different locations. Considering evidence on deprivations is a way of supplementing the information available on the money metric poverty lines to provide a more comprehensive account of child well-being (Minujin and Nandy, 2012). In addition, deprivation studies are likely to be closer to the reality of people’s perceptions of poverty and well-being (Chambers et al., 2001).

A feature of the MICS is that it collected information on the ownership of household goods and amenities. Items included were electricity supply, radio, TV, mobile phone, static phone, refrigerator, watch, bicycle, motorcycle, cart, car, motorized boat, canoe, source of drinking water and type of sanitary facility. Additional household characteristics and circumstances were also included: the number of persons per sleeping room, type of floor, type of roof, type of wall and type of cooking fuel.

## Levels of deprivation

Table 7 shows the childhood experience of either ‘severe’ or ‘less severe’ deprivations. With the exception of information deprivation (51%), severe deprivations were relatively uncommon, ranging between 3% for severe sanitation deprivation, and 5% for severe education deprivation, to 17% for health deprivation. However, significant proportions of the population under age 17 experienced less severe deprivations, particularly health (65%), information (55%), shelter (44%) and sanitation (38%). Children appeared to experience less severe deprivations in the areas of food, water and education.

**Table 7.** Children experiencing severe and less severe levels of deprivation.

Deprivation	Severe %	Less severe %
**Shelter**	14	44
**Sanitation**	3	38
**Water**	8	16
**Information**	51	55
**Education**	5	23
**Food**	10	26
**Health**	17	65

## Multiple deprivations

An important issue is whether children experience multiple deprivations of basic human need for shelter, sanitation, safe water, information, health, education and food. Table 8 shows the experience of multiple deprivations in Vanuatu, it is evident that a significant proportion of children in Vanuatu suffered from severe deprivation (one or more severe deprivations) of basic human need. Over half of all children (52%) experienced at least one severe deprivation and close to four-fifths (76%) experienced at least one less severe deprivation. Nearly a fifth (16%) of children in Vanuatu suffered from absolute poverty (two or more severe deprivations) and over half (52%) experienced two or more less severe deprivations of human need.

**Table 8.** Children’s experiences of multiple deprivations.

	Severe %	Less severe %
**One or more deprivations**	52.1	76.1
**Two or more deprivations**	16.4	52.4
**Three or more deprivations**	3.4	27.4
**Four or more deprivations**	0.5	8.7
**Five or more deprivations**	0.0	1.0

Table 9 shows the nature of severe deprivations by region. Torba experienced the highest percentage of severe deprivation of all regions, primarily because of its poor performance on the measure of shelter deprivation, with almost half of all households having coral or earth floors. Torba, like most other regions, fares poorly on health measures, and food deprivation at 7.9% is just below the rural average.

**Table 9.** Prevalence of severe childhood deprivations by province and residence.

Region	Shelter %	Sanitation %	Water %	Information %	Food %	Education %	Health %
**Torba**	41.6	4.6	5.6	81.7	7.9	10.4	18.2
**Sanma**	10.7	9.2	10.9	63.0	14.1	2.8	19.2
**Penama**	37.3	0.0	5.5	73.6	8.1	6.8	18.9
**Malampa**	3.3	0.0	3.9	66.4	6.9	0.7	2.9
**Shefa**	0.5	0.5	4.4	34.1	5.0	3.7	16.7
**Tafea Luganville**	29.5	7.9	21.3	69.8	7.5	12.8	22.5
	3.2	3.1	0.0	12.5	11.4	3.1	26.1
**Port Vila**	0.9	0.5	1.3	5.9	19.6	4.3	24.6
**Residence**							
**Urban**	1.5	1.2	0.9	7.7	17.4	4.0	25.0
**Rural**	16.7	3.7	9.2	62.4	8.1	5.5	15.4

Arguably the worst-off province was Tafea, which fared poorly on shelter, sanitation, water, education and health, although food deprivation was just below the rural average. In rural areas, Sanma fares worst on food poverty. Penama was another region characterized by poor shelter conditions. Urban areas, particularly Port Vila, had the highest incidence of food deprivation. Health deprivations were also higher in both urban areas, although the rural average was reduced by the low level of health deprivation in Malampa.

After information and shelter, health was the most frequent deprivation. All regions, with the exception of Malampa, experienced high levels of health deprivation. The MICS3 survey confirms low overall immunization rates – with only 42% of Vanuatu children under 2 being fully immunized. Only a quarter of children had full immunization by the time they were 1 year old. This situation is far below the target adopted by Vanuatu to reach 90% national coverage of full immunization.

Mobile telephone network coverage has improved since the time of the MICS3 survey and so information deprivation may be declining, particularly in rural areas, with infrastructure investment (Pacific Institute of Public Policy, 2009). That said however, mobile network coverage alone does not necessarily guarantee poor families access to telephone services and therefore we cannot speculate on the impact of this initiative in the absence of more reliable information at the household level.

### Relative poverty lines

Poverty lines expressed as a percentage of median income or expenditure are widely used to compare poverty rates and levels of inequality, in Europe and North America, for example, but are now being applied in developing country contexts (Esser and William, 2013). Many argue that household expenditure is preferable to household income for poverty measurement, as consumption expenditure represents the day-to-day purchases and is usually closer to household consumption (Canberra Group, 2011; Rio Group, 2006). The use of relative poverty lines, here at 50% and 60% of the household median, can be viewed as a method of bringing inequality into the discussion of poverty, since poverty rates will be higher in countries where low-income groups are at a greater distance from the median.

In Table 10, we observe that nearly 25% of Vanuatu children lived in households with expenditure less than 50% of the national median and just under a third with expenditure less than 60% of the national median. Tafea, Shefa and Penama experienced high ‘absolute’ and ‘basic needs’ poverty rates, but relative poverty rates were highest in Torba, with 45% of children living below 50% of the national median and 57% living below 60% of the national median.

**Table 10.** Children in poverty using median equivalized household consumption expenditure.

Region	Total No. children	Below 50% of median	Below 60% of median
**Torba**	4,420	45.7	56.9
**Sanma (rural)**	11,673	19.2	29.5
**Penama**	15,059	21.5	28.2
**Malampa**	13,081	17.8	26.5
**Shefa (rural)**	12,067	27.0	34.8
**Tafea**	15,786	35.2	43.8
**Luganville**	4,408	9.0	14.0
**Port Vila**	11,492	7.9	11.0
**Vanuatu**	**87,986**	**22.7**	**30.3**

## Discussion

In summary, a number of conclusions can be drawn from the poverty and deprivation estimates presented here. Methodologically, the results show that measurement issues are extremely important and that different approaches can have a substantial impact on the level of poverty and deprivation measured and identified. Table 11 summarizes our results, which suggest that 5% of all children in Vanuatu live in poverty, as defined by the international dollar a day measure. A much greater proportion, 17%, live in poverty defined by the national food and basic needs poverty line, or 11% if needs are priced at the regional level; 16% of children suffer from absolute poverty (two or more serve deprivations), 23% live in households with income below 50% of the median and 30% with income below 60% of the median. Therefore, on the basis of our study, we suggest that the current international poverty line of a dollar a day seriously underestimates global poverty levels. In the context of Vanuata, our triangulated results suggest underestimation of poverty by at least a third (or over 10,000 people) in the population aged 17 years or under. Thus, the tighter definition of poverty used by the World Bank tends to lead to a better-looking poverty trend, because the poverty line is too low the trend it reports is too rosy.

**Table 11.** Child poverty estimates in Vanuata.

	Estimated prevalence of poverty %
**$1.25 a-day Poverty Line**	5.4
**National Food and Basic Needs Poverty Line**	17.0
**Regional Food and Basic Needs Poverty Line**	11.0
**Absolute Poverty (two or more severe deprivations)**	16.4
**50% median income**	22.7
**60% median income**	30.3

Further issues for discussion concern the generalizability of our study findings and their potential implications for the measurement of global poverty. We observe a growing body of empirical research scholarship using basic needs and caloric standards that similarly exposes the underestimation of extreme global poverty defined by the World Bank dollar a day measure, particularly in the Latin American context where recent work has suggested poverty rates twice the level of the World Bank estimates (ECLAC, 2002, 2011). The inadequacies of the international poverty line of a dollar a day continue to be exposed. The standard is increasingly seen as being arbitrary since it is not closely related to the consumption or expenditure or income needed to avoid extreme poverty, nor is it closely related to calorific requirements or demographic characteristics (Reddy and Pogge, 2010; Townsend, 2002). We may conclude then, if the World Bank had in fact used a poverty line grounded in basic needs rather than its present artificial one the total number of poor people in world would increase substantially, perhaps by as much as 30% as this inquiry and the growing body of empirical evidence suggests (Wade, 2007). This is because the basic needs (food, clothes, shelter) poverty lines and the severe deprivation poverty measure reported here indicate such an increase.

In order to assess how our different poverty measures compare we examine the relationships in the regional results using correlation techniques. We took the average regional prevalence of severe childhood deprivations from Table 9, and correlated this with the regional results for the US dollar a day line (Table 3), and the regional results for the national BNPL (Table 5). The correlation coefficients were 0.47 and 0.72 respectively. Thus, the national BNPL money metric has the highest correlation with deprivation poverty at the national level, and thus we suggest provides the more reliable estimate of severe poverty compared to the World Bank dollar a day.^7^

Deprivations and poverty may also have a very strong regional dimension within national states. Importantly, we found that drawing a BNPL at the national level yields significantly different results from setting a line varying by region. Such differences are to be expected – this is because the standard population used to calculate the poverty lines has shifted from the national level to the regional level. Port Vila, for example, moves from near the bottom of the poverty ranking to near the top when the sub-national BNPL is used. The poverty experienced in Port Vila is likely to be related to its much higher cost of living compared with the rest of country. The analysis of deprivations also shows that disparities in child well-being have an important locational dimension. Whichever measure is used, however, children living in the most remote provinces consistently experience high poverty rates.

## Measurement issues

It is now widely accepted in social policy that using more than one measure of poverty is often the best approach to identify those at risk of poverty (Bradshaw and Finch, 2003; Rio Group, 2006). In this article we have considered a range of monetary and deprivation measures of poverty, including income inequality measures employed in high-income settings. We find that the different approaches offer complementary ways of gathering information about family and household living standards. Each method captures different groups of children, for whom interventions may need to be different. At present, we are unable to link the data reliably from our two datasets (i.e. linking household budget survey data with the household deprivation survey) in order to examine levels of consistent poverty and deprivation. This would be desirable, however. National statistical agencies and NGOs, the World Bank, and other philanthropic agencies and donors (e.g. UNICEF) should give increasing attention to these sorts of survey design issues. Better surveys and/or data linkages would facilitate more valid and reliable measurement and identification of consistent poverty and deprivation.

Non-household groups and populations also continue to be an issue for poverty measurement. The estimates presented here, and those used by the World Bank in Table 1, are likely to underestimate the scale and nature of the global poverty problem by not capturing non-household populations. In developing countries, population estimates and assessments of progress towards the MDGs are based increasingly on household surveys. As Carr-Hill (2012) argues, household surveys are often inappropriate or inadequate for obtaining information about the poorest of the poor people. This is because they typically omit by design people who do not live in households, either because they are homeless or they live in institutions. Often, there are also issues with mobile and/or nomadic populations. In practice, it is difficult for surveys to reach those in fragile, temporary, disjointed or multiple-occupancy households. Survey researchers may also struggle to reach people living in urban slums or in areas where there may be security risks.

## Social policies

Extreme poverty rates, as shown in Table 1, are down from 1.94 billion people living in poverty in 1981 to 1.29 billion people in 2008, and have fallen by half from the 1990 MDG baseline. Nevertheless, current projections indicate that, by 2015, almost 1 billion people will still be living on less than $1.25 per day. Reducing the number of people living on less than $1.25 a day to zero, therefore, continues to be one of the most pressing global priorities of our age. The disturbing fact is that this initiative will not end severe poverty or hardship. That is because the dollar a day measure is an inadequate measure of poverty on which to be base a global poverty eradication strategy, as it clearly fails to identify people living in severe poverty and deprivation. And, while much more can be done to boost private sector investment opportunities in countries like Vanuatu, as the Asian Development Bank (ADB) claims, the development of the finance and service sectors, and infrastructure projects, would contribute to the overarching objective of poverty reduction (ADB, 2009). However, we may also wish to draw attention to some of the more fundamental problems with current neoliberal policy prescriptions. ‘Washington Consensus’ policies, it is argued, are failing to make a real impact on chronic global poverty and deprivation – at best progress has been slow (Anand et al., 2010; Townsend, 2009). Much of the reduction in extreme poverty, seen in Table 1, took place in China. Progress elsewhere, over nearly three decades, has been stubbornly slow, particularly in sub-Saharan Africa, casting a long shadow of doubt over the ‘evidence base’ supporting the present global policy response to extreme poverty and deprivation (CSDH, 2008). By contrast, a broad coalition of bodies under the United Nations recommends a global social protection floor to tackle the problem of chronic global poverty (ILO, 2011), which would provide support, by means of unconditional cash transfers, to those families not covered by adequate social security and living in extreme poverty. The recommendation for a basic right to income security for all is a breakthrough in global social policy. National social protection floors can be a major tool to achieve the targets of the United Nations’ MDGs (ILO, 2012). Clearly, there is more pressing work to be done to build comprehensive social security systems and adequate national social protection floors to provide decent living standards for all beyond a dollar a day.

## Notes

1. UNICEF provides a working definition of child poverty, which, inspired by the principles of the Convention on the Rights of the Child, focuses on the resources children need to survive and grow: ‘Children living in poverty experience deprivation of the material, spiritual, and emotional resources needed to survive, develop and thrive, leaving them unable to enjoy their rights, achieve their full potential or participate as full and equal members of society’ (UNICEF, 2005: 15).

2. A High-Level Panel on the Post-2015 Development Agenda was set up by the United Nations that will now shape a new global approach to international development.

3. See ‘World Bank Group President Calls for a World Free of Poverty’ (2 April 2013), at http://www.worldbank.org/en/news/press-release/2013/04/02/world-bank-group-president-calls-world-free-poverty (viewed June 2013).

4. The HDI is a composite statistic used to rank countries’ achievements in three basic aspects of human development relating to life expectancy, education and income indices. The HDI composite statistic ranges from 0, the lowest score, to 1, the highest score. The value for Vanuatu in 2012 was 0.626, for Australia it is 0.938.

5. The minimum food-energy intake recommended by the Food and Agricultural Organization (FAO) of the United Nations, and the World Health Organization (WHO).

6. The UNDP equivalence scale gives the same weight to all adults in a household, but treats each child as half an adult.

7. A high degree of correlation between the measures is not altogether surprising since they are all measuring the same thing (i.e. poverty) – and it would be alarming if there was no relationship at all between them. On the other hand, we would not expect to find them perfectly correlated as they are very different measures of poverty and they tap into different aspects and dimensions of poverty. As a result, they are likely to produce different estimates on the prevalence of poverty, as our study findings suggest.
